# A Gastric Glycoform of MUC5AC Is a Biomarker of Mucinous Cysts of the Pancreas

**DOI:** 10.1371/journal.pone.0167070

**Published:** 2016-12-19

**Authors:** Jessica Sinha, Zheng Cao, Jianliang Dai, Huiyuan Tang, Katie Partyka, Galen Hostetter, Diane M. Simeone, Ziding Feng, Peter J. Allen, Randall E. Brand, Brian B. Haab

**Affiliations:** 1 Van Andel Research Institute, Grand Rapids, MI, United States of America; 2 MD Anderson Cancer Center, Houston, TX, United States of America; 3 University of Michigan School of Medicine, Ann Arbor, MI, United States of America; 4 Memorial Sloan Kettering Cancer Center, New York, NY, United States of America; 5 University of Pittsburgh Medical Center, Pittsburgh, PA, United States of America; Centro Nacional de Investigaciones Oncologicas, SPAIN

## Abstract

Molecular indicators to specify the risk posed by a pancreatic cyst would benefit patients. Previously we showed that most cancer-precursor cysts, termed mucinous cysts, produce abnormal glycoforms of the proteins MUC5AC and endorepellin. Here we sought to validate the glycoforms as a biomarker of mucinous cysts and to specify the oligosaccharide linkages that characterize MUC5AC. We hypothesized that mucinous cysts secrete MUC5AC displaying terminal N-acetylglucosamine (GlcNAc) in either alpha or beta linkage. We used antibody-lectin sandwich assays to detect glycoforms of MUC5AC and endorepellin in cyst fluid samples from three independent cohorts of 49, 32, and 66 patients, and we used monoclonal antibodies to test for terminal, alpha-linked GlcNAc and the enzyme that produces it. A biomarker panel comprising the previously-identified glycoforms of MUC5AC and endorepellin gave 96%, 96%, and 87% accuracy for identifying mucinous cysts in the three cohorts with an average sensitivity of 92% and an average specificity of 94%. Glycan analysis showed that MUC5AC produced by a subset of mucinous cysts displays terminal alpha-GlcNAc, a motif expressed in stomach glands. The alpha-linked glycoform of MUC5AC was unique to intraductal papillary mucinous neoplasms (IPMN), whereas terminal beta-linked GlcNAc was increased in both IPMNs and mucinous cystic neoplasms (MCN). The enzyme that synthesizes alpha-GlcNAc, A4GNT, was expressed in the epithelia of mucinous cysts that expressed alpha-GlcNAc, especially in regions with high-grade dysplasia. Thus IPMNs secrete a gastric glycoform of MUC5AC that displays terminal alpha-GlcNAc, and the combined alpha-GlcNAc and beta-GlcNAc glycoforms form an accurate biomarker of mucinous cysts.

## Introduction

Cysts of the pancreas develop through various pathways. Some cysts arise from the accumulation of fluid or debris from a nearby lesion, but other cysts—termed true cysts—form from the neoplastic growth of epithelial cells that secrete fluid into an enclosed lumen [1, 2]. Certain sub-types of neoplastic cysts can progress into pancreatic cancer given the right combination of mutations and microenvironmental factors. Studies of genetically-engineered mouse models [3, 4] and retrospective analyses of human cysts suggest that pancreatic cancers arising from cysts are essentially equivalent to adenocarcinomas arising from solid precursor lesions. Not all neoplastic cysts, however, have the potential to progress to cancer. Those that may progress to cancer include intraductal papillary mucinous neoplasms (IPMNs), which typically harbor mutations in the GNAS, KRAS and RNF43 genes [5] and form in the main and branch ducts of the pancreas; mucinous cystic neoplasms (MCNs), which display mutations in RNF43 and KRAS and are typically solitary lesions that occur in the body or tail of the pancreas [5, 6]; and solid pseudopapillary neoplasms (SPNs), which show mutations in the CTNNB1 (beta-catenin) gene [5]. Serous cystadenomas display mutations in VHL and are considered benign as they typically do not progress to cancer [5, 6].

In the clinical evaluation of a cyst, the first goal in order to guide management is to determine if the cyst is a cancer precursor [7]. This distinction may be difficult. The images of cysts obtained by endoscopic ultrasound and other modalities are only 50–73% accurate in differentiating mucinous from non-mucinous cysts [8–10] and require significant expertise in the observer. The level of CEA in the cyst fluid may help distinguish mucinous from non-mucinous cysts [11], but this test leaves much ambiguity. A recent study found a sensitivity of 61% and specificity of 77%, resulting in a misclassification of 39% of mucinous cases [12], which is similar to previous findings [11, 13, 14]. The lack of clarity has produced much debate about optimal indications for surgical resection [15], with the goal of balancing the potentially positive outcome from removing a dangerous lesion against the negative consequences of an unnecessary procedure. Among patients having surgery for a pancreatic cyst, 20–60% are found to have non-mucinous cysts [16–18].

Cyst fluid, which can be sampled by fine-needle aspiration, is a proximal and potentially rich source for biomarkers. Many candidate biomarkers in the cyst fluid are under investigation (reviewed in [19, 20]), including microRNAs [21, 22], metabolites [23], and inflammatory cytokines [24]. The analysis of mutations in DNA found in cyst fluid [5, 6] or ductal secretions [25] may be particularly valuable for diagnosis of the cyst type, although assays for clinical use are not yet validated. A promising test for the positive identification of serous cystadenomas is VEGF, which was elevated in 17/17 serous cases and only 1/24 mucinous cysts [26].

We previously presented the discovery and initial validation of a panel of biomarkers in the cyst fluid that accurately distinguishes mucinous from non-mucinous cysts [27, 28]. The 3-marker panel, composed of specific glycoforms of the proteins MUC5AC and endorepellin, performed better than carcinoembryonic antigen (CEA). Therefore, our first goal in this study was to perform additional blinded studies to determine if the panel would be valuable in the clinical evaluation of pancreatic cysts. A second goal was to specify the oligosaccharide linkages that define the abnormal glycosylation. Previous analyses suggested the presence of terminal N-acetylglucosamine (GlcNAc) [28], an unusual motif not normally seen in the pancreas, but we did not have definitive confirmation of this motif, and we did not know whether the linkage of the GlcNAc was in the alpha or the beta confirmation. The latter distinction carries biological information, as the two linkages arise in different cell types and have different interaction partners.

We demonstrate that specific glycoforms of MUC5AC and endorepellin form an accurate biomarker of mucinous cysts, and that the glycoforms of MUC5AC in mucinous cysts display two unusual motifs, terminal αGlcNAc and terminal βGlcNAc. The two motifs appear in distinct groups of patients, with the αGlcNAc version exclusively in IPMNs and potentially produced at elevated levels in dysplastic epithelia with high-grade dysplasia. The two glycoforms represent novel molecular markers of mucinous cysts and together yield accurate discrimination of mucinous from non-mucinous cysts.

## Materials and Methods

### Human Pancreatic Cyst Fluid Specimens

The study was conducted under protocols approved by the Institutional Review Boards at the Van Andel Research Institute, the University of Pittsburgh Medical Center, Memorial Sloan Kettering Cancer Center, and the University of Michigan Medical School. All subjects provided written, informed consent.

Cyst fluid samples were collected at the University of Pittsburgh Medical Center, Memorial Sloan-Kettering Cancer Center, and the University of Michigan Medical Center (Table 1 and S1 Table). All samples were collected by either endoscopic ultrasound-guided, fine-needle aspiration (EUS-FNA) or FNA from surgically removed cysts. We previously verified that the method of collecting the cyst fluid does not greatly affect specific marker levels [29]. The samples were collected prior to diagnosis, and the subjects were selected for the study without regard to diagnosis. The study included subjects that 1) underwent surgical removal of a pancreatic cyst; consented to the study; and had available cyst fluid. No subjects were excluded for insufficient volume of cyst fluid. The surgical pathology report confirmed the histological diagnosis of cyst type in all subjects in the study.

**Table 1:** Sample cohorts.

	Cohort 1	Cohort 2	Cohort 3	Total
Total Samples	49	32	66	147
Mucinous	25 (51%)	26 (81%)	55 (83%)	106
Type				
IPMN	17	20	35	72
MCN	8	6	19	33
Other	-	-	1	1
Collection Site				
UM	1	0	0	1
UPMC	22	26	0	48
MSKCC	2	0	55	57
Non-mucinous	24 (49%)	6 (19%)	11 (17%)	41
Type				
PC	17	-	-	17
SC	2	2	11	15
NET	5	3	-	8
Retention	-	1	-	1
Collection Site				
UM	1	0	0	1
UPMC	22	6	0	28
MSKCC	1	0	11	12

IPMN, intraductal papillary mucinous neoplasm; MCN, mucinous cystic neoplasm; PC, pseudocyst; SC, serous cystadenoma; NET, neuroendocrine tumor; UM, University of Michigan; UPMC, University of Pittsburgh Medical Center; MSKCC, Memorial Sloan Kettering Cancer Center.

The specimens were kept on ice until aliquots were made and frozen at -80°C, within 2 h of collection. An aliquot that had been thawed no more than twice was used for each experiment. Prior to each experiment, we removed the cellular debris and clot fragments from each aliquot by centrifuging the sample for 10 min at 2,400 x g and collecting the supernatant.

### Antibody-Lectin Sandwich Array Experiments

The antibodies, lectins, control proteins, and enzymes were purchased from various sources (S2 Table). The buffers and biological solutions used in the microarray assays were: 1X phosphate-buffered saline with 0.5% or 0.1% Tween-20 (referred to as PBST0.5 or PBST0.1); 1X PBS with 1% Tween-20 and 1% Brij-35 (Thermo Scientific, Rockford, IL) (referred to as 10X sample buffer); 1X PBS with 400 μg/mL each of mouse, sheep, and goat IgG, and 800 μg/mL rabbit IgG (antibodies from Jackson Immunoresearch) (referred to as 4X IgG blocking cocktail); 1X PBS with 1 tablet protease inhibitor (Complete Tablet, Roche Applied Science, Indianapolis, IN) per 1 mL PBS (referred to as 10X protease inhibitor); and 1X PBS with 2X sample buffer, 2X protease inhibitor, and 2X IgG cocktail (referred to as 2X sample dilution buffer).

The capture antibodies were spotted onto coated microscope slides (PATH, Grace Bio-Labs, Bend, OR) using a robotic arrayer (2470, Aushon Biosystems, Billerica, MA). The antibodies were prepared at 250 μg/mL in 1X PBS with 0.005% Tween-20 and 15% glycerol. Each slide contained 48 identical arrays arranged in a 4x12 grid with 4.5 mm spacing between arrays, and each array had the same antibodies printed in six-replicate. A wax hydrophobic border was imprinted (SlideImprinter, The Gel Company, San Francisco, CA) to define boundaries between the arrays [30, 31]. The printed slides were stored at 4°C in a desiccated, vacuum-sealed slide box until use.

The antibody-lectin sandwich assays were modified from the protocol described previously [27]. The cyst fluid samples were diluted 4-fold into the sample buffer (1 part cyst fluid, 2 parts 2X sample buffer, and 1 part 1X PBS) and incubated overnight at 4°C with gentle agitation to allow for blocking of non-specific binding to the added IgG in the sample buffer. The next day, the slides were blocked with 1% bovine serum albumin (BSA, Fisher Scientific, Fair Lawn, NJ) in PBST0.5 for 1 hour, washed in three changes of PBST0.5 for 3 min each, and dried by brief centrifugation at 160 x g.

We incubated 6 μL of each diluted sample on an array for 2 hours at RT, or overnight at 4°C for larger experiments, and we applied each sample to 3 replicate arrays. After sample incubation, the slides were washed three times in PBST0.1 and spin-dried. Next the captured antigens were detected for 1 hour with biotinylated antibodies or lectins (3 μg/mL) prepared in 0.1% BSA/PBST0.1, followed by 1 hour incubation with Cy5-conjugated streptavidin (43–4316, Invitrogen, Carlsbad, CA) at 2 μg/ml in 0.1% BSA/PBST0.1 with three washes between steps. Lastly, all the spin-dried slides were scanned for fluorescence at 633 nm using a microarray scanner (LS Reloaded, TECAN, Morrisville, NC).

The resulting images were quantified and analyzed using custom, in-house software [32] that locates the spots and quantifies their intensities, subtracts the local background from the median intensity of each spot, and removes any outliers from the six-replicate spots. To remove outliers, the program calculates the Grubbs’ statistic for the spot farthest from the mean of the replicates, rejects the spot if the Grubbs’ statistic exceeds a preset threshold (here using p < 0.1), and repeatedly removes spots until no outliers remain or to a minimum of three spots. It then calculates the geometric mean of the remaining replicate spots for each array, and finally calculates the mean and coefficient of variation between replicate arrays for each sample. All samples sets were run at least twice in entirety, and the correlations across samples between replicate sets were 0.90–0.95 (not shown).

### Immunohistochemistry

The VARI Biospecimen facility provided formalin-fixed, paraffin-embedded tissue from patients who underwent resections of pancreatic cysts at regional hospitals in Grand Rapids, MI. The Institutional Review Board at the Van Andel Research Institute approved this research project.

We used automated staining (Ventana Discovery Ultra) to perform immunohistochemistry on 5 μm tissue sections from formalin-fixed, paraffin-embedded blocks. The protocol included antigen retrieval using the Ventana CC1 buffer for 36 minutes at 95°C; anti-A4GNT (HPA008017, Sigma) incubation at a 1:25 dilution for 32 minutes at 37°C; and secondary antibody for 12 minutes at 37°C. The development step used the diaminobenzadine chromagen according to preset parameters in the Ventana platform. We acquired images of the slides with the Aperio ScanScope XT.

### Data and Figure Preparation

The data were analyzed and prepared using Microsoft Office Excel, OriginPro 8 (OriginLab, Northampton, MA), and MedCalc 12.3.0.0 (MedCalc Software, Mariakerke, Belgium). We prepared the figures using Canvas 14 (ACD Systems).

## Results

### Validation of Candidate Biomarkers

**Fig 1:**
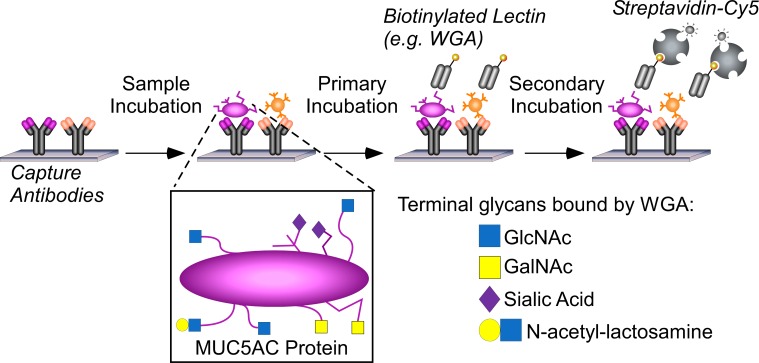
Measuring protein glycoforms in clinical samples. Antibodies immobilized on coated glass slides capture specific proteins out of cyst fluid samples, and lectins or glycan-binding antibodies probe glycans on the captured proteins. We apply each sample to multiple arrays in parallel and probe each array with its own glycan-binding reagent. Some lectins such as wheat-germ agglutinin (WGA) bind several glycan motifs, leaving ambiguity about which motifs are elevated in particular samples.

Our first goal was to validate a previously-defined biomarker panel for the differentiation of mucinous from non-mucinous pancreatic cysts. The panel consisted of the glycoforms of the proteins MUC5AC and endorepellin detected by the lectin wheat-germ agglutinin (notated MUC5AC:WGA and endorepellin:WGA, respectively) and the glycoform of MUC5AC detected by an antibody against the blood group H antigen (MUC5AC:BGH). We determined the levels of the markers by antibody-lectin sandwich arrays (Fig 1).

We obtained samples in 3 separate cohorts (Table 1). Cohorts 1 and 2 were from the University of Pittsburgh Medical Center (except for 2 samples from the University of Michigan), and Cohort 3 was from Memorial Sloan Kettering Cancer Center. After obtaining measurements of the individual biomarkers in all samples, we classified each sample using a combination rule that was defined previously [28]. If a sample showed an elevation in MUC5AC:WGA or in at least two of the three biomarkers, we classified the sample as a mucinous cyst; otherwise we classified it as a non-mucinous cyst. The thresholds defining elevations were determined from samples analyzed previously.

**Fig 2:**
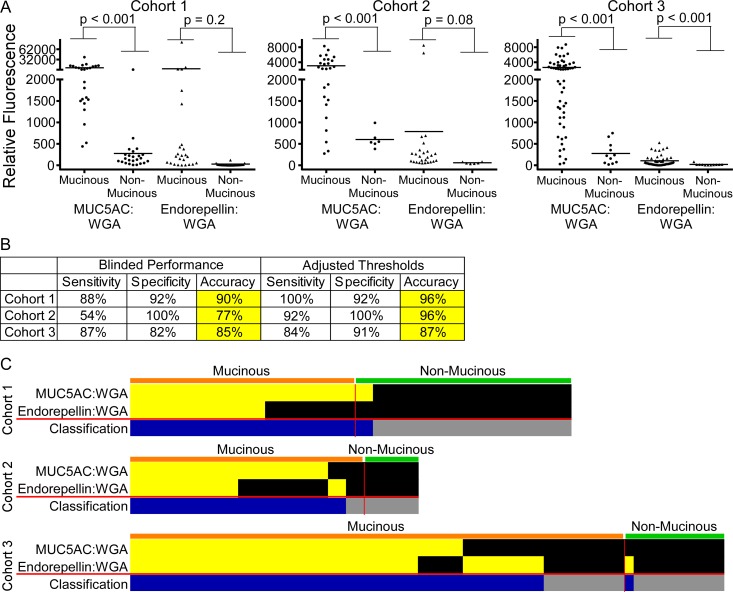
Validation of a previously-discovered biomarker panel. A) The individual biomarkers had significantly higher levels in mucinous cysts relative to non-mucinous cysts in all 3 cohorts. B) The combination of the two biomarkers shown in panel A distinguished mucinous from non-mucinous cysts with high accuracy. After determining the initial performance in each cohort, we adjusted the individual marker thresholds. The sensitivity in cohort 2 improved markedly after adjustment. C) The individual biomarkers showed elevations in non-identical mucinous cysts, rendering the panel more accurate than either marker used alone. Yellow boxes indicate measurements that were above a threshold for a particular marker. In the bottom row, blue boxes indicate samples with an elevation in either marker, classified as mucinous.

We performed the experiments blinded to the diagnoses associated with cohorts 1 and 2 until after the initial testing of the panel. We ran cohort 3 as if blinded, in that we randomized the samples and made classifications solely based on pre-defined rules. The individual biomarkers were significantly elevated in the mucinous cysts relative to the non-mucinous cysts (Fig 2A), especially MUC5AC:WGA. The MUC5AC:BGH marker (not shown) was only weakly significant and did not contribute to the panel performance in all three cohorts.

The panel of biomarkers, whereby a sample with an elevation in any marker was classified as mucinous, achieved 90% and 85% accuracies in cohorts 1 and 3, respectively, and a lower accuracy of 77% in cohort 2, mainly due to low sensitivity (54%) (Fig 2B). This performance was consistent with the previous results. The thresholds defining elevations were derived from limited cohort sizes in the previous studies [27, 28], so it was possible that adjustments to the thresholds would improve performance. After unblinding the diagnoses and adjusting the thresholds for each marker, we increased the performance slightly for cohorts 1 and 3 and considerably for cohort 2 (Fig 2B). Thus the markers and combination rule are effective, but further optimization of the thresholds based on more patient data may be necessary.

MUC5AC:WGA accounted for most of the elevations, although endorepellin:WGA contributed 7 unique elevations in the cases and 1 false-positive elevation in cohort 3 (Fig 2C). The MUC5AC:WGA and endorepellin:WGA biomarkers were not statistically different between various types of mucinous cysts, including IPMN relative to MCN, and high-grade dysplasia relative to low-grade dysplasia (not shown). We also found that the cyst fluid biomarkers were rarely secreted into the blood circulation. In matched plasma samples from 42 of the patients, only 4 had detectable levels of MUC5AC:WGA (not shown).

### αGlcNAc Expression in the Cyst Fluid

Because WGA binds several different glycan motifs (Fig 1), we sought to more precisely characterize the glycoform elevated in mucinous cysts. Our previous study indicated the primary motifs elevated in mucinous cysts could be terminal N-acetylglucosamine (GlcNAc) or N-acetylgalactosamine (GalNAc) [28], so we focused on differentiating between these two motifs and between their alpha-linked and beta-linked variants.

We tested for terminal αGlcNAc using a monoclonal antibody [33]; for terminal βGlcNAc using a lectin from *Sclerotium rolfsii* (SRL); and for both terminal αGlcNAc and terminal alpha-N-acetyl-galactosamine (αGalNAc) using a lectin from *Helix aspersa* (HAA). Glycan array analysis (S1 Fig) shows the binding of WGA to three glycan motifs, αGlcNAc, βGlcNAc, and αGalNAc; binding of SRL to αGlcNAc and β GlcNAc; and binding of HAA to αGlcNAc and αGalNAc.

**Fig 3:**
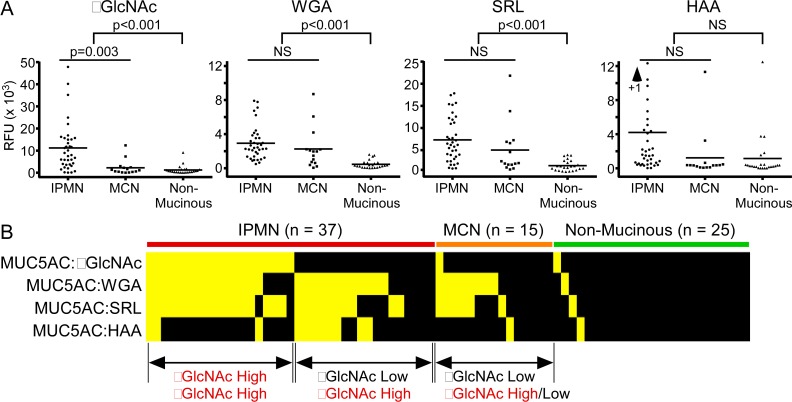
Elevation of the αGlcNAc and βGlcNAc glycoforms of MUC5AC in mucinous cysts. A) The αGlcNAc glycoform of MUC5AC was higher in mucinous relative to non-mucinous cysts, and in IPMNs relative to MCNs. The WGA-reactive and SRL-reactive glycoforms were higher in mucinous cysts but not in IPMNs relative to MCNs. The HAA-reactive glycoform was on average higher in IPMNs but not statistically significantly (by t-test) due to high variability between individual levels. B) About half of the IPMNs were elevated in αGlcNAc, and most of the remaining IPMNs were elevated in βGlcNAc. The MCNs are not elevated in αGlcNAc, and about half are elevated in βGlcNAc.

Using 77 samples collated from Cohorts 1–3, we found a significant elevation in the αGlcNAc glycoform of MUC5AC in the fluid of mucinous cysts relative to non-mucinous cysts (Fig 3A). The glycoforms of MUC5AC detected by WGA, SRL, and HAA also were elevated in mucinous cysts, but only the αGlcNAc glycoform was significantly elevated in IPMNs relative to MCNs (Fig 3A). A view of all markers together (Fig 3B) showed that the IPMNs fall into two groups: those elevated in both αGlcNAc and β GlcNAc, and those elevated in β GlcNAc only. The glycoform detected by HAA (which binds both αGlcNAc and αGalNAc) does not correlate with the glycoform detected by the other reagents, confirming the presence of terminal αGlcNAc in a unique subset of IPMNs.

### A4GNT and αGlcNAc Expression in Tissue

**Fig 4:**
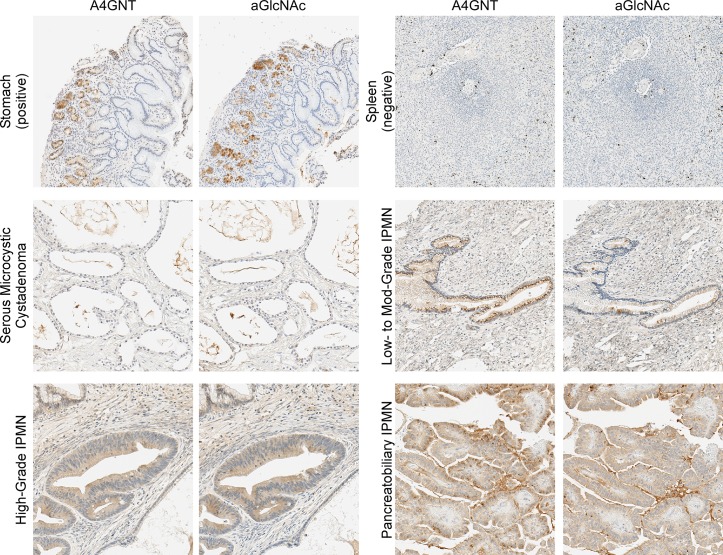
Correlated expression of A4GNT and αGlcNAc in the dysplastic epithelia of mucinous cysts. We stained adjacent sections of each block with anti-A4GNT or anti-αGlcNAc. The 2 antibodies showed strong similarity in their staining. Glandular cells of the stomach were strongly and mulitfocally positive. Spleen cells were negative with the exception of scattered resident macrophages. The staining of cystic epithelia was low in a non-mucinous cyst and high in mucinous cysts, particularly in high-grade dysplasia and pancreatobiliary IPMN.

We next investigated the cellular origin of αGlcNAc expression in pancreatic cysts. The terminal αGlcNAc motif is produced in the stomach epithelia and liver hepatocytes by the enzyme alpha-1,4-N-acetylglucosaminyltransferase (A4GNT) [34, 35]. We confirmed that both the A4GNT and αGlcNAc antibodies stained glandular cells in stomach epithelia but not negative control tissue (Fig 4A and 4B). The 2 antibodies showed remarkably similar staining in adjacent sections from a variety of cystic tissue (not from the same patients as the cyst fluid samples). Tissue from a non-mucinous serous cyst was negative, but epithelia from IPMNs with low-to-high grade dysplasia were positive in both antibodies. The subcellular localizations in certain regions are different between the antibodies, perhaps because the enzyme is not always producing the αGlcNAc epitope, but generally the staining appears in the same areas. The A4GNT staining is both diffusely cytoplasmic and punctate, but we do not yet have an explanation for the variation in staining pattern. Selected regions from 5 different mucinous cysts confirmed A4GNT in neoplastic cysts with particularly strong staining in epithelia with high-grade dysplasia (S2 Fig). Thus these findings support the concept that αGlcNAc is produced by A4GNT in the dysplastic epithelia of mucinous cysts.

## Discussion

The performance of the biomarker panel comprising the WGA-reactive glycoforms of MUC5AC and endorepellin, averaging 92% sensitivity and 94% specificity, compares favorably with recent results on potential biomarkers of mucinous cysts. Among other approaches under investigation, a promising area is the analysis of DNA mutations in the cyst fluid. Early studies focused on the detection of KRAS mutations and loss-of-heterozygosity, which showed good specificity but limited sensitivity (45%) for identifying malignant and premalignant cysts [36]. Because GNAS mutations appear in some mucinous cysts that are negative for KRAS mutations, a test evaluating both mutations performs better than tests evaluating either mutation individually. A test for a mutation in either KRAS or GNAS gave 65% sensitivity and 100% specificity [37] for identifying mucinous differentiation, with higher sensitivity for identifying just IPMNs. Performance can be further enhanced if evaluating clinical and imaging features with GNAS or KRAS mutations. Such an approach performed with 90–100% sensitivity and 92–98% specificity to classify cyst type [38], although researchers have not yet performed a blinded analysis. Other mutations among a panel of 39 cancer-related genes do not further add to performance over GNAS and KRAS [39]. A potential limitation of DNA analysis is that the sensitivities are low. The glycoform biomarkers reported here had an average sensitivity of 92% with a specificity of 94%. If the glycoforms are present in the mucinous cysts that do not show DNA mutations, the combination of the glycoforms with DNA mutations could achieve very good sensitivity while maintaining high specificity. Additional studies will be required to examine the combination markers and to further validate the use of the novel MUC5AC glycoforms as biomarkers.

The limitations of this study are that it was performed on subjects for whom pathological confirmation of diagnosis was available, thus not completely representing the patient population; it used an assay that was not established in a clinical laboratory; and some of the samples were collected on the surgical specimen, rather than via fine-needle aspiration in the diagnostic setting. But these limitations are necessary in the current phase of research and are common among studies of potential biomarkers in cyst fluid. Future work will involve specifying the molecules that provide the most information about cyst diagnosis and prognosis; transferring the assays to a clinical platform; and conducting prospective studies on their clinical use. In particular, an important question is whether separately measuring αGlcNAc and βGlcNAc is more informative than a measuring them together in a single assay, as with WGA.

A clear implication of this study is that the epithelial cells in mucinous cysts alter the glycoforms of their secreted and membrane-bound proteins as they become neoplastic. Pancreatic cysts have a tendency to take on altered morphologies that reflect other tissue types [40], so the presence of αGlcNAc could indicate transdifferentiation of some sort. A previous study demonstrated positive staining for αGlcNAc in IPMNs mainly of the gastric type, suggesting its use as a marker of gastric differentiation [41], but we also observed it in IPMNs with intestinal differentiation. Future studies should further examine the origin and functions of the αGlcNAc epitope. One of the functions of αGlcNAc in the stomach appears to be protection against infection by pathogens such as *H*. *pylori* through the inhibition of an enzyme critical for pathogen survival [42, 43], and it also may help suppress cancer-inducing inflammation [44], but its role in IPMNs may be something else. An interesting question is whether the cells producing the beta-linked version are different from the cells that produce the alpha-linked version, and if so, whether these glycoforms represent markers for subtypes of IPMNs. Additional studies could be directed toward identifying cellular differentiation markers that co-express with either αGlcNAc or βGlcNAc, and then to investigate the behaviors of each cell type. Increased knowledge about the cellular changes that accompany progression toward malignancy, and about differences between individual cases, could lead to more accurate diagnoses and prognoses of mucinous cysts.

In addition to differentiating mucinous from non-mucinous cysts, a valuable cyst fluid test would indicate the potential of malignancy. The current guidelines leave much uncertainty, particularly for branch-duct IPMNs that typically are not resected yet nevertheless pose a threat to the patient [45]. DNA mutations do not provide information about the threat of malignancy, nor do other molecular biomarkers, although inflammatory cytokines such as IL-1β show promise [24]. The best predictors of malignancy remain clinical and imaging factors [46]. The histomorphologic subtype is associated with outcome—cysts with pancreatobiliary differentiation have worse prognosis [47]—but such cell characteristics cannot be ascertained by cytological examination of cyst fluid. Furthermore, the identification of pathologic subtype based on morphology and immunohistochemistry can be uncertain in up to 25% of cases [48], even if whole sections are available. Potentially a glycoform could give such information, given the importance of cell-surface glycans in the biology of epithelia. Future studies could examine whether particular glycans such as αGlcNAc accurately indicate prognosis or the presence of high-grade dysplasia.

## Conclusions

IPMNs produce a gastric glycoform of MUC5AC that displays terminal αGlcNAc, and MCNs and most IPMNs produce another unusual glycoform of MUC5AC that displays terminal βGlcNAc. A panel comprising these two glycoforms of MUC5AC and a specific glycoform of endorepellin is an accurate biomarker of mucinous cysts. Additional studies will address that possibility that these biomarkers indicate biological distinctions between mucinous cysts that have value for guiding treatment.

## Supporting Information

S1 Fig Glycan array analysis of lectin specificities. (PDF)

S2 Fig A4GNT is elevated in the dysplastic epithelia of pancreatic cysts. (PDF)

S1 Table Patient sample information. (PDF)

S2 Table Reagent information. (PDF)

## Abbreviations

WGA: wheat germ agglutinin
MCN: mucinous cystic neoplasm
IPMN: intraductal papillary mucinous neoplasm
PC: pseudocyst
SC: serous cystadenoma
GlcNAc: N-acetylglucosamine
GalNAc: N-acetylgalactosamine

## References

[1] Singh M, Maitra A. Precursor lesions of pancreatic cancer: molecular pathology and clinical implications. Pancreatology. 2007;7(1):9–19. 10.1159/000101873 17449961

[2] Basturk O, Coban I, Adsay NV. Pancreatic cysts: pathologic classification, differential diagnosis, and clinical implications. Arch Pathol Lab Med. 2009;133(3):423–38. Epub 2009/03/06. 10.1043/1543-2165-133.3.423 19260748

[3] Siveke JT, Einwachter H, Sipos B, Lubeseder-Martellato C, Kloppel G, Schmid RM. Concomitant pancreatic activation of Kras(G12D) and Tgfa results in cystic papillary neoplasms reminiscent of human IPMN. Cancer cell. 2007;12(3):266–79. 10.1016/j.ccr.2007.08.002 17785207

[4] Izeradjene K, Combs C, Best M, Gopinathan A, Wagner A, Grady WM, et al Kras(G12D) and Smad4/Dpc4 haploinsufficiency cooperate to induce mucinous cystic neoplasms and invasive adenocarcinoma of the pancreas. Cancer cell. 2007;11(3):229–43. 10.1016/j.ccr.2007.01.017 17349581

[5] Wu J, Jiao Y, Dal Molin M, Maitra A, de Wilde RF, Wood LD, et al Whole-exome sequencing of neoplastic cysts of the pancreas reveals recurrent mutations in components of ubiquitin-dependent pathways. Proceedings of the National Academy of Sciences of the United States of America. 2011;108:21188–93. Epub 2011/12/14. 10.1073/pnas.1118046108 22158988 PMC3248495

[6] Wu J, Matthaei H, Maitra A, Dal Molin M, Wood LD, Eshleman JR, et al Recurrent GNAS Mutations Define an Unexpected Pathway for Pancreatic Cyst Development. Sci Transl Med. 2011;3(92):92ra66 Epub 2011/07/22. 10.1126/scitranslmed.3002543 21775669 PMC3160649

[7] Maitra A, Fukushima N, Takaori K, Hruban RH. Precursors to invasive pancreatic cancer. Advances in anatomic pathology. 2005;12(2):81–91. 15731576 10.1097/01.pap.0000155055.14238.25

[8] Fisher WE, Hodges SE, Yagnik V, Moron FE, Wu MF, Hilsenbeck SG, et al Accuracy of CT in predicting malignant potential of cystic pancreatic neoplasms. HPB (Oxford). 2008;10(6):483–90. Epub 2008/12/18. PubMed Central PMCID: PMCPMC2597321. 19088937 10.1080/13651820802291225 PMC2597321

[9] Ahmad NA, Kochman ML, Lewis JD, Ginsberg GG. Can EUS alone differentiate between malignant and benign cystic lesions of the pancreas? The American journal of gastroenterology. 2001;96(12):3295–300. Epub 2002/01/05. 10.1111/j.1572-0241.2001.05328.x 11774939

[10] Frossard JL, Amouyal P, Amouyal G, Palazzo L, Amaris J, Soldan M, et al Performance of endosonography-guided fine needle aspiration and biopsy in the diagnosis of pancreatic cystic lesions. The American journal of gastroenterology. 2003;98(7):1516–24. Epub 2003/07/23. 10.1111/j.1572-0241.2003.07530.x 12873573

[11] Brugge WR, Lewandrowski K, Lee-Lewandrowski E, Centeno BA, Szydlo T, Regan S, et al Diagnosis of pancreatic cystic neoplasms: a report of the cooperative pancreatic cyst study. Gastroenterology. 2004;126(5):1330–6. Epub 2004/05/08. 15131794 10.1053/j.gastro.2004.02.013

[12] Gaddam S, Ge PS, Keach JW, Mullady D, Fukami N, Edmundowicz SA, et al Suboptimal accuracy of carcinoembryonic antigen in differentiation of mucinous and nonmucinous pancreatic cysts: results of a large multicenter study. Gastrointestinal endoscopy. 2015. Epub 2015/06/17. 10.1016/j.gie.2015.04.040 26077458

[13] van der Waaij LA, van Dullemen HM, Porte RJ. Cyst fluid analysis in the differential diagnosis of pancreatic cystic lesions: a pooled analysis. Gastrointestinal endoscopy. 2005;62(3):383–9. 16111956 10.1016/s0016-5107(05)01581-6

[14] Nagula S, Kennedy T, Schattner MA, Brennan MF, Gerdes H, Markowitz AJ, et al Evaluation of Cyst Fluid CEA Analysis in the Diagnosis of Mucinous Cysts of the Pancreas. J Gastrointest Surg. 2010;12:1997–2003. Epub 2010/07/27. 10.1007/s11605-010-1281-0 20658204

[15] Sahora K, Mino-Kenudson M, Brugge W, Thayer SP, Ferrone CR, Sahani D, et al Branch duct intraductal papillary mucinous neoplasms: does cyst size change the tip of the scale? A critical analysis of the revised international consensus guidelines in a large single-institutional series. Annals of surgery. 2013;258(3):466–75. Epub 2013/09/12. 10.1097/SLA.0b013e3182a18f48 24022439

[16] Cho CS, Russ AJ, Loeffler AG, Rettammel RJ, Oudheusden G, Winslow ER, et al Preoperative classification of pancreatic cystic neoplasms: the clinical significance of diagnostic inaccuracy. Annals of Surgical Oncology. 2013;20(9):3112–9. Epub 2013/04/19. 10.1245/s10434-013-2986-6 23595223 PMC4857772

[17] Nadig SN, Pedrosa I, Goldsmith JD, Callery MP, Vollmer CM. Clinical implications of mucinous nonneoplastic cysts of the pancreas. Pancreas. 2012;41(3):441–6. Epub 2011/10/22. 10.1097/MPA.0b013e318229b9b8 22015974

[18] Raty S, Sand J, Laukkarinen J, Vasama K, Bassi C, Salvia R, et al Cyst fluid SPINK1 may help to differentiate benign and potentially malignant cystic pancreatic lesions. Pancreatology. 2013;13(5):530–3. Epub 2013/10/01. 10.1016/j.pan.2013.06.008 24075519

[19] Rockacy M, Khalid A. Update on pancreatic cyst fluid analysis. Annals of gastroenterology: quarterly publication of the Hellenic Society of Gastroenterology. 2013;26(2):122–7. PubMed Central PMCID: PMC3959935. PMC3959935 24714589

[20] Thiruvengadam N, Park WG. Systematic Review of Pancreatic Cyst Fluid Biomarkers: The Path Forward. Clinical and translational gastroenterology. 2015;6:e88 Epub 2015/06/13. 10.1038/ctg.2015.17 26065716 PMC4816245

[21] Matthaei HH, Wylie D, Lloyd MB, Dal Molin M, Kemppainen J, Mayo SC, et al MicroRNA biomarkers in cyst fluid augment the diagnosis and management of pancreatic cysts. Clinical cancer research: an official journal of the American Association for Cancer Research. 2012. Epub 2012/06/23. 10.1158/1078-0432.CCR-12-0035 PMC3547600 22723372

[22] Ryu JK, Matthaei H, Dal Molin M, Hong SM, Canto MI, Schulick RD, et al Elevated microRNA miR-21 Levels in Pancreatic Cyst Fluid Are Predictive of Mucinous Precursor Lesions of Ductal Adenocarcinoma. Pancreatology. 2011;11(3):343–50. Epub 2011/07/16. 10.1159/000329183 21757972 PMC3142103

[23] Park WG, Wu M, Bowen R, Zheng M, Fitch WL, Pai RK, et al Metabolomic-derived novel cyst fluid biomarkers for pancreatic cysts: glucose and kynurenine. Gastrointestinal endoscopy. 2013. Epub 2013/04/10. 10.1016/j.gie.2013.02.037 PMC3780566 23566642

[24] Maker AV, Katabi N, Qin LX, Klimstra DS, Schattner M, Brennan MF, et al Cyst fluid interleukin-1beta (IL1beta) levels predict the risk of carcinoma in intraductal papillary mucinous neoplasms of the pancreas. Clin Cancer Res. 2011;17(6):1502–8. Epub 2011/01/27. PubMed Central PMCID: PMC3065716. 10.1158/1078-0432.CCR-10-1561 21266527 PMC3065716

[25] Kanda M, Knight S, Topazian M, Syngal S, Farrell J, Lee J, et al Mutant GNAS detected in duodenal collections of secretin-stimulated pancreatic juice indicates the presence or emergence of pancreatic cysts. Gut. 2013;62(7):1024–33. Epub 2012/08/04. 10.1136/gutjnl-2012-302823 22859495 PMC3893110

[26] Yip-Schneider MT, Wu H, Dumas RP, Hancock BA, Agaram N, Radovich M, et al Vascular endothelial growth factor, a novel and highly accurate pancreatic fluid biomarker for serous pancreatic cysts. J Am Coll Surg. 2014;218(4):608–17. Epub 2014/02/05. 10.1016/j.jamcollsurg.2013.12.019 24491241

[27] Haab BB, Porter A, Yue T, Li L, Scheiman J, Anderson MA, et al Glycosylation Variants of Mucins and CEACAMs as Candidate Biomarkers for the Diagnosis of Pancreatic Cystic Neoplasms. Annals of surgery. 2010;251(5):937–45. 10.1097/SLA.0b013e3181d7738d 20395854 PMC3713623

[28] Cao Z, Maupin K, Curnutte B, Fallon B, Feasley CL, Brouhard E, et al Specific glycoforms of MUC5AC and endorepellin accurately distinguish mucinous from nonmucinous pancreatic cysts. Mol Cell Proteomics. 2013;12(10):2724–34. Epub 2013/07/10. PubMed Central PMCID: PMCPMC3790286. 10.1074/mcp.M113.030700 23836919 PMC3790286

[29] Partyka K, McDonald M, Maupin KA, Brand R, Kwon R, Simeone DM, et al Comparison of surgical and endoscopic sample collection for pancreatic cyst fluid biomarker identification. Journal of proteome research. 2012;11(5):2904–11. Epub 2012/03/24. PubMed Central PMCID: PMC3345068. 10.1021/pr2012736 22439797 PMC3345068

[30] Forrester S, Kuick R, Hung KE, Kucherlapati R, Haab BB. Low-volume, high-throughput sandwich immunoassays for profiling plasma proteins in mice: identification of early-stage systemic inflammation in a mouse model of intestinal cancer. Molecular Oncology. 2007;1:216–25. 10.1016/j.molonc.2007.06.001 19305640 PMC2658882

[31] Haab BB, Yue T. High-throughput studies of protein glycoforms using antibody-lectin sandwich arrays. Methods in molecular biology (Clifton, NJ. 2011;785:223–36. Epub 2011/09/09. 10.1007/978-1-61779-286-1_15 PMC3705222 21901603

[32] Ensink E, Sinha J, Sinha A, Tang H, Calderone HM, Hostetter G, et al Segment and Fit Thresholding: A New Method for Image Analysis Applied to Microarray and Immunofluorescence Data. Analytical chemistry. 2015;87(19):9715–21. Epub 2015/09/05. 10.1021/acs.analchem.5b03159 26339978 PMC4854282

[33] Nakamura N, Ota H, Katsuyama T, Akamatsu T, Ishihara K, Kurihara M, et al Histochemical reactivity of normal, metaplastic, and neoplastic tissues to alpha-linked N-acetylglucosamine residue-specific monoclonal antibody HIK1083. J Histochem Cytochem. 1998;46(7):793–801. Epub 1998/06/20. 9632738 10.1177/002215549804600702

[34] Nakayama J, Yeh JC, Misra AK, Ito S, Katsuyama T, Fukuda M. Expression cloning of a human alpha1, 4-N-acetylglucosaminyltransferase that forms GlcNAcalpha1—>4Galbeta—>R, a glycan specifically expressed in the gastric gland mucous cell-type mucin. Proceedings of the National Academy of Sciences of the United States of America. 1999;96(16):8991–6. Epub 1999/08/04. PubMed Central PMCID: PMCPMC17720. 10430883 10.1073/pnas.96.16.8991 PMC17720

[35] Zhang MX, Nakayama J, Hidaka E, Kubota S, Yan J, Ota H, et al Immunohistochemical demonstration of alpha1,4-N-acetylglucosaminyltransferase that forms GlcNAcalpha1,4Galbeta residues in human gastrointestinal mucosa. J Histochem Cytochem. 2001;49(5):587–96. Epub 2001/04/17. 11304796 10.1177/002215540104900505

[36] Khalid A, Zahid M, Finkelstein SD, LeBlanc JK, Kaushik N, Ahmad N, et al Pancreatic cyst fluid DNA analysis in evaluating pancreatic cysts: a report of the PANDA study. Gastrointestinal endoscopy. 2009;69(6):1095–102. Epub 2009/01/21. 10.1016/j.gie.2008.07.033 19152896

[37] Singhi AD, Nikiforova MN, Fasanella KE, McGrath KM, Pai RK, Ohori NP, et al Preoperative GNAS and KRAS testing in the diagnosis of pancreatic mucinous cysts. Clin Cancer Res. 2014;20(16):4381–9. Epub 2014/06/19. 10.1158/1078-0432.CCR-14-0513 24938521

[38] Springer S, Wang Y, Dal Molin M, Masica DL, Jiao Y, Kinde I, et al A Combination of Molecular Markers and Clinical Features Improve the Classification of Pancreatic Cysts. Gastroenterology. 2015;149(6):1501–10. Epub 2015/08/09. 10.1053/j.gastro.2015.07.041 26253305 PMC4782782

[39] Jones M, Zheng Z, Wang J, Dudley J, Albanese E, Kadayifci A, et al Impact of next-generation sequencing on the clinical diagnosis of pancreatic cysts. Gastrointestinal endoscopy. 2015. Epub 2015/08/09. 10.1016/j.gie.2015.06.047 26253016

[40] Adsay NV, Merati K, Basturk O, Iacobuzio-Donahue C, Levi E, Cheng JD, et al Pathologically and biologically distinct types of epithelium in intraductal papillary mucinous neoplasms: delineation of an "intestinal" pathway of carcinogenesis in the pancreas. The American journal of surgical pathology. 2004;28(7):839–48. 15223952 10.1097/00000478-200407000-00001

[41] Kobayashi M, Fujinaga Y, Ota H. Reappraisal of the Immunophenotype of Pancreatic Intraductal Papillary Mucinous Neoplasms (IPMNs)-Gastric Pyloric and Small Intestinal Immunophenotype Expression in Gastric and Intestinal Type IPMNs. Acta histochemica et cytochemica. 2014;47(2):45–57. Epub 2014/09/16. PubMed Central PMCID: PMCPMC4138401. 10.1267/ahc.13027 25221363 PMC4138401

[42] Kawakubo M, Ito Y, Okimura Y, Kobayashi M, Sakura K, Kasama S, et al Natural antibiotic function of a human gastric mucin against Helicobacter pylori infection. Science (New York, NY. 2004;305(5686):1003–6. Epub 2004/08/18. 10.1126/science.1099250 15310903

[43] Ito Y, Vela JL, Matsumura F, Hoshino H, Tyznik A, Lee H, et al Helicobacter pylori cholesteryl alpha-glucosides contribute to its pathogenicity and immune response by natural killer T cells. PLoS One. 2013;8(12):e78191 Epub 2013/12/07. PubMed Central PMCID: PMCPMC3846475. 10.1371/journal.pone.0078191 24312443 PMC3846475

[44] Karasawa F, Shiota A, Goso Y, Kobayashi M, Sato Y, Masumoto J, et al Essential role of gastric gland mucin in preventing gastric cancer in mice. The Journal of clinical investigation. 2012;122(3):923–34. Epub 2012/02/07. PubMed Central PMCID: PMC3287219. 10.1172/JCI59087 22307328 PMC3287219

[45] Fritz S, Klauss M, Bergmann F, Hackert T, Hartwig W, Strobel O, et al Small (Sendai negative) branch-duct IPMNs: not harmless. Annals of surgery. 2012;256(2):313–20. Epub 2012/07/14. 10.1097/SLA.0b013e31825d355f 22791105

[46] Correa-Gallego C, Do R, Lafemina J, Gonen M, D'Angelica MI, DeMatteo RP, et al Predicting dysplasia and invasive carcinoma in intraductal papillary mucinous neoplasms of the pancreas: development of a preoperative nomogram. Annals of Surgical Oncology. 2013;20(13):4348–55. Epub 2013/09/21. 10.1245/s10434-013-3207-z 24046103

[47] Distler M, Kersting S, Niedergethmann M, Aust DE, Franz M, Ruckert F, et al Pathohistological Subtype Predicts Survival in Patients With Intraductal Papillary Mucinous Neoplasm (IPMN) of the Pancreas. Annals of surgery. 2013. Epub 2013/03/28. 10.1097/SLA.0b013e318287ab73 23532107

[48] Schaberg KB, DiMaio MA, Longacre TA. Intraductal Papillary Mucinous Neoplasms Often Contain Epithelium From Multiple Subtypes and/or Are Unclassifiable. The American journal of surgical pathology. 2015. 10.1097/PAS.0000000000000528 26469398

