# Supplementary material for: A Gastric Glycoform of MUC5AC Is a Biomarker of Mucinous Cysts of the Pancreas
Source: PLoS One. 2016 Dec 19;11(12):e0167070. doi: 10.1371/journal.pone.0167070 (PMC5167232; doi:10.1371/journal.pone.0167070)
Supplement: S1 Fig — (PDF) [file pone.0167070.s001.pdf]

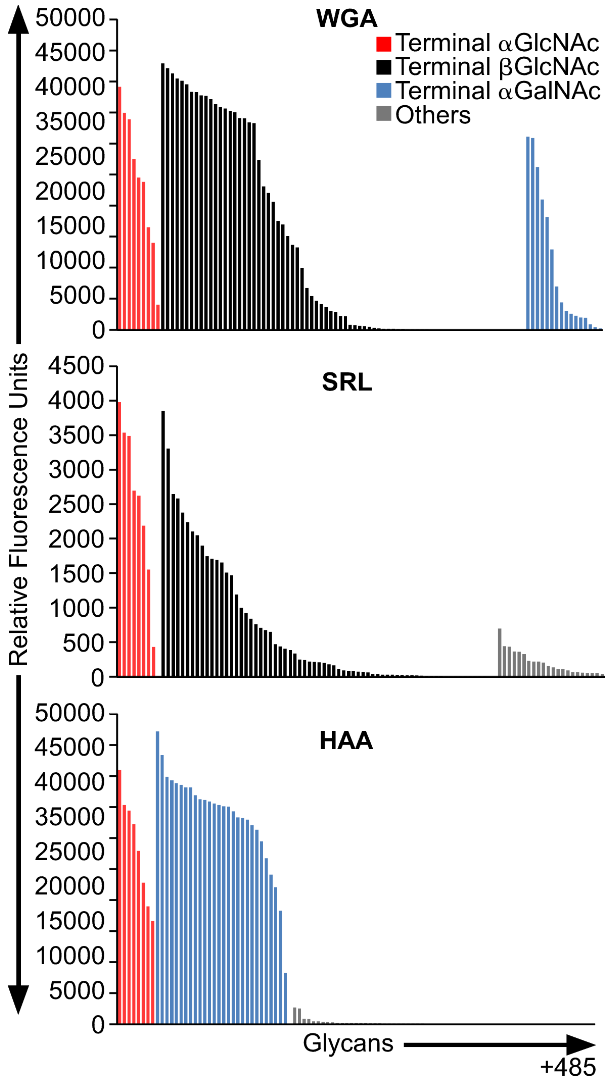

S1 Fig. Glycan array analysis of lectin specificities. The glycan synthesis and array core facility of the Consortium for Functional Glycomics (CFG) performed the glycan array experiments and the primary analysis. We downloaded data from www.functionalglycomics.org that previously had been obtained using lectins and glycan-binding antibodies supplied by various investigators. For detailed analyses of the datasets, we used the Motif Segregation method{porter haab 2010} and the GlycoSearch analysis program (23). The graphs show the fluorescence intensity of various glycans, grouped as indicated by the legend. All 3 lectins share binding to αGlcNAc (red bars). WGA, wheat-germ agglutinin; SRL, *Sclerotium rolfsii* lectin; HAA, *Helix aspersa* agglutinin; GlcNAc, N-acetyl-glucosamine; GalNAc, N-acetyl-galactosamine.
