# Supplementary material for: A Gastric Glycoform of MUC5AC Is a Biomarker of Mucinous Cysts of the Pancreas
Source: PLoS One. 2016 Dec 19;11(12):e0167070. doi: 10.1371/journal.pone.0167070 (PMC5167232; doi:10.1371/journal.pone.0167070)
Supplement: S2 Fig — (PDF) [file pone.0167070.s002.pdf]

A

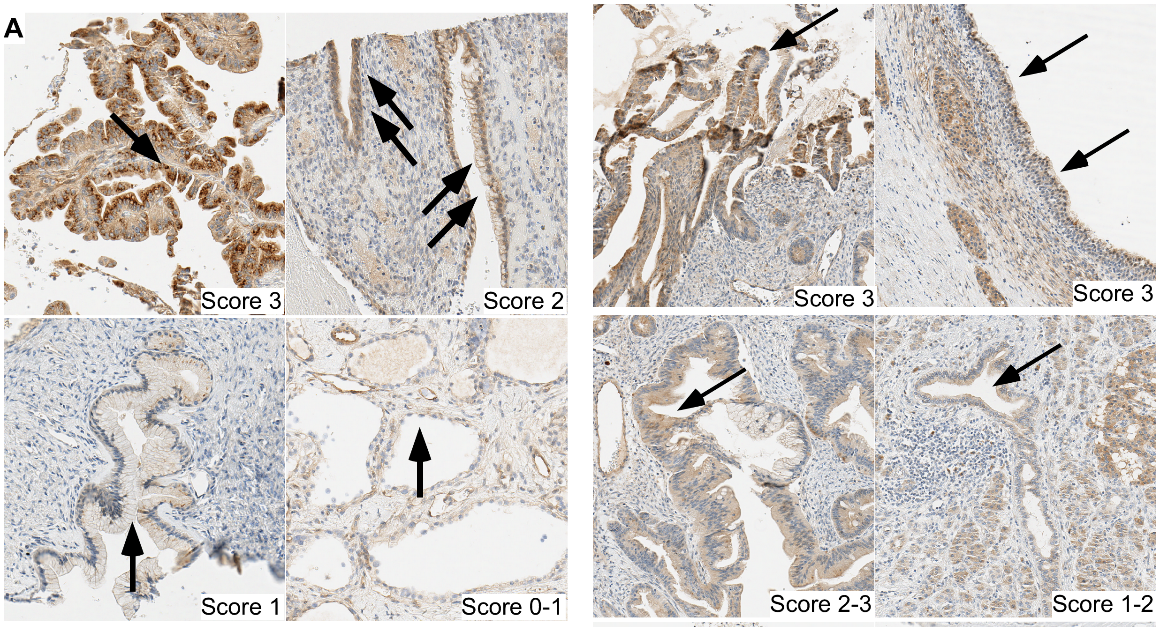

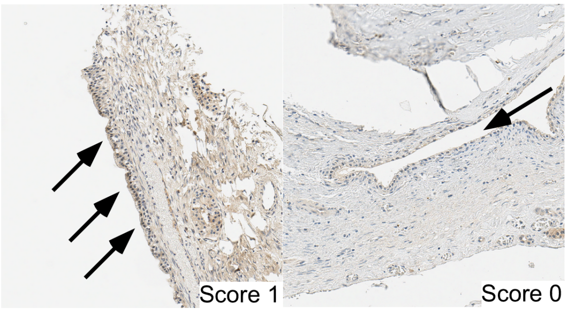

B

	Grading		
	**Normal/Low-Grade**	**Mod-/High-Grade**	
**Score 0-1**	21	16	37
**Score 2-3**	7	26	33
	28	42	70

p=0.003

S2 Fig. A4GNT is elevated in the dysplastic epithelia of pancreatic cysts. A) We performed immunohistochemistry for A4GNT expression in 6 sections from 5 different IPMNs and 1 section from a serous microcystic cystadenoma. A pathologist (Dr. Hostetter) scored the level of dysplasia and the level of staining in 77 different regions containing epithelia, of equal size and evenly distributed between the cases. The figure displays representative regions, and the arrows indicate representative epithelial cells that were scored. B) An analysis by Fisher's Exact test of the association between dysplasia grade and staining intensity showed a statistically significant relationship.
