# Supplementary material for: A Gastric Glycoform of MUC5AC Is a Biomarker of Mucinous Cysts of the Pancreas
Source: PLoS One. 2016 Dec 19;11(12):e0167070. doi: 10.1371/journal.pone.0167070 (PMC5167232; doi:10.1371/journal.pone.0167070)
Supplement: S1 Table — (PDF) [file pone.0167070.s003.pdf]

S1 Table. Patient Sample Information. IPMN, intraductal papillary mucinous neoplasm; MCN, mucinous cystic neoplasm; SC, serous cystadenoma; PC, pseudocyst; PNET, pancreatic neuroendocrine tumor

Cohort	Final set	Classification	Cyst Type	Collection Site	Diagnosis	Histological Type	Grade
0	1	Mucinous	IPMN	MSKCC	IPMN		
0	1	Mucinous	cancer	U. Michigan	Microscopic foci of invasive	Microscopic foci of invasive	
0	1	Mucinous	MCN	U. Michigan	MCN		
0	1	Non-mucinous	SC	U. Michigan	SC		
0	1	Non-mucinous	SC	U. Michigan	SC		
1	0	Mucinous	IPMN	MSKCC	IPMN		
1	1	Non-mucinous	PNET	MSKCC	PNET		
1	0	Mucinous	MCN	MSKCC	cancer		
1	0	Mucinous	MCN	U. Michigan	MCN		
1	0	Non-mucinous	PC	U. Michigan	PC		
1	0	Mucinous	IPMN	UPMC	IPMN (surgical)		adenoCA
1	0	Mucinous	IPMN	UPMC	IPMN (surgical)		Low
1	0	Mucinous	IPMN	UPMC	IPMN (surgical)		Mod
1	1	Mucinous	IPMN	UPMC	IPMN (surgical)		Mod
1	0	Mucinous	IPMN	UPMC	IPMN (surgical)		Mod
1	0	Mucinous	IPMN	UPMC	IPMN (surgical)		Low
1	1	Mucinous	IPMN	UPMC	IPMN (surgical)		Mod
1	1	Mucinous	IPMN	UPMC	IPMN (surgical)		Low
1	0	Mucinous	IPMN	UPMC	IPMN (surgical)		Low
1	0	Mucinous	IPMN	UPMC	IPMN (surgical)		Low
1	0	Mucinous	IPMN	UPMC	IPMN (surgical)		Mod
1	1	Mucinous	IPMN	UPMC	IPMN (surgical)		Mod
1	0	Mucinous	IPMN	UPMC	IPMN (surgical)		High
1	0	Mucinous	IPMN	UPMC	IPMN (surgical)		adenoCA
1	0	Mucinous	IPMN	UPMC	IPMN (surgical)		adenoCA
1	0	Mucinous	IPMN	UPMC	IPMN (surgical)		adenoCA
1	1	Mucinous	MCN	UPMC	MCN (surgical)		Low
1	0	Mucinous	MCN	UPMC	MCN (surgical)		Low
1	0	Mucinous	MCN	UPMC	MCN (surgical)		Low
1	0	Mucinous	MCN	UPMC	MCN (surgical)		High
1	1	Mucinous	MCN	UPMC	MCN (surgical)		Low
1	1	Mucinous	MCN	UPMC	MCN (surgical)		Low
1	0	Non-mucinous	PC	UPMC	pseudocyst		
1	1	Non-mucinous	PC	UPMC	pseudocyst		
1	0	Non-mucinous	PC	UPMC	pseudocyst		
1	1	Non-mucinous	PC	UPMC	pseudocyst		
1	1	Non-mucinous	PC	UPMC	pseudocyst		
1	0	Non-mucinous	PC	UPMC	pseudocyst		
1	1	Non-mucinous	PC	UPMC	pseudocyst		
1	1	Non-mucinous	PC	UPMC	pseudocyst		
1	1	Non-mucinous	PC	UPMC	pseudocyst		
1	1	Non-mucinous	PC	UPMC	pseudocyst		
1	1	Non-mucinous	PC	UPMC	pseudocyst		
1	1	Non-mucinous	PC	UPMC	pseudocyst		
1	1	Non-mucinous	PC	UPMC	pseudocyst		
1	1	Non-mucinous	PC	UPMC	pseudocyst		
1	1	Non-mucinous	PC	UPMC	pseudocyst		
1	0	Non-mucinous	PC	UPMC	pseudocyst		
1	0	Non-mucinous	PNET	UPMC	neuoendocrine (surgical)		
1	0	Non-mucinous	PNET	UPMC	neuroendocrine (surgical)		
1	0	Non-mucinous	PNET	UPMC	neuroendocrine (surgical)		
1	0	Non-mucinous	PNET	UPMC	neuroendocrine		High
1	0	Non-mucinous	SC	UPMC	SC (CLIN)		
1	0	Non-mucinous	SC	UPMC	SC (surgical)		
2	0	Mucinous	IPMN	UPMC	IPMN (surgical)		
2	0	Mucinous	IPMN	UPMC	IPMN (surgical)		
2	0	Mucinous	IPMN	UPMC	IPMN (surgical)		
2	0	Mucinous	IPMN	UPMC	IPMN (surgical)		
2	0	Mucinous	IPMN	UPMC	IPMN (surgical)		
2	0	Mucinous	IPMN	UPMC	IPMN (surgical)		
2	0	Mucinous	IPMN	UPMC	IPMN (surgical)		
2	0	Mucinous	IPMN	UPMC	IPMN (surgical)		
2	0	Mucinous	IPMN	UPMC	IPMN (surgical)		
2	1	Mucinous	IPMN	UPMC	IPMN degenerated into adenocarcinoma		
2	0	Mucinous	IPMN	UPMC	IPMN (surgical)		
2	1	Mucinous	IPMN	UPMC	IPMN degenerated into adenocarcinoma		
2	0	Mucinous	IPMN	UPMC	IPMN (surgical)		

2	0	Mucinous	IPMN	UPMC	IPMN (surgical)		
2	0	Mucinous	IPMN	UPMC	IPMN (surgical)		
2	0	Mucinous	IPMN	UPMC	IPMN (surgical)		
2	0	Mucinous	IPMN	UPMC	IPMN (surgical)		
2	0	Mucinous	IPMN	UPMC	panc adenocarcinoma/IPMN (surgical)		
2	0	Mucinous	IPMN	UPMC	IPMN (surgical)		
2	1	Mucinous	IPMN	UPMC	IPMN degenerated into adenocarcinoma		
2	0	Mucinous	MCN	UPMC	muc cyst adenoma (surgical)		
2	0	Mucinous	MCN	UPMC	muc cyst adenoma (surgical)		
2	0	Mucinous	MCN	UPMC	muc cyst adenoma (surgical)		
2	0	Mucinous	MCN	UPMC	muc cyst adenoma (surgical)		
2	0	Mucinous	MCN	UPMC	muc cyst adenoma (surgical)		
2	1	Mucinous	MCN	UPMC	muc cyst adenoma (surgical)		
2	0	NE	Neuroendocrine	UPMC	neuroendocrine tumor		
2	0	NE	Neuroendocrine	UPMC	neuroendocrine tumor		
2	0	NE	Neuroendocrine	UPMC	neuroendocrine tumor		
2	0	Non-mucinous	Retention	UPMC	benign retention cyst (surgical)		
2	1	Non-mucinous	SC	UPMC	serous cystadenoma (surgical)		
2	1	Non-mucinous	SC	UPMC	serous cystadenoma (surgical)		
3	0	Mucinous	IPMN	MSKCC	IPMN, main duct	Carcinoma; intestinal	High
3	1	Mucinous	IPMN	MSKCC	IPMN		Mod
3	1	Mucinous	IPMN	MSKCC	IPMN, branch duct		Low
3	1	Mucinous	IPMN	MSKCC	IPMN, main duct	invasive ductal adenocarcinoma	High
3	1	Mucinous	IPMN	MSKCC	IPMN, main duct		Mod
3	1	Mucinous	IPMN	MSKCC	IPMN, branch duct	gastric type	Low
3	1	Mucinous	IPMN	MSKCC	IPMN, main duct	intestinal type	Mod
3	1	Mucinous	IPMN	MSKCC	IPMN, branch duct		Mod
3	1	Mucinous	IPMN	MSKCC	IPMN, main duct	intestinal type	Mod
3	1	Mucinous	IPMN	MSKCC	IPMN		Mod
3	1	Mucinous	IPMN	MSKCC	IPMN, mixed main/branch duct	gastric type	Mod
3	1	Mucinous	IPMN	MSKCC	IPMN	gastric type	Mod
3	0	Mucinous	IPMN	MSKCC	IPMN	gastric type	Mod
3	1	Mucinous	IPMN	MSKCC	IPMN		Mod
3	1	Mucinous	IPMN	MSKCC	IPMN, branch duct	branch duct	Mod
3	1	Mucinous	IPMN	MSKCC	IPMN, mixed main/branch duct	carcinoma	High
3	0	Mucinous	IPMN	MSKCC	IPMN, branch duct		Mod
3	1	Mucinous	IPMN	MSKCC	IPMN, branch duct		Mod
3	1	Mucinous	IPMN	MSKCC	IPMN, carcinoma; main duct	mixed gastric/panc type	High
3	0	Mucinous	IPMN	MSKCC	IPMN, branch duct type		Mod
3	1	Mucinous	MCN	MSKCC	MCN		Low
3	1	Mucinous	MCN	MSKCC	MCN		Low
3	1	Mucinous	MCN	MSKCC	MCN		Low
3	1	Mucinous	MCN	MSKCC	MCN		Low
3	1	Mucinous	MCN	MSKCC	MCN		Low
3	1	Mucinous	MCN	MSKCC	MCN		Low
3	1	Mucinous	MCN	MSKCC	MCN		Mod
3	1	Mucinous	MCN	MSKCC	MCN		Low
3	1	Mucinous	MCN	MSKCC	MCN		Mod
3	0	Mucinous	MCN	MSKCC	MCN		Mod
3	1	Mucinous	MCN	MSKCC	MCN		Low
3	0	Mucinous	MCN	MSKCC	MCN		Low
3	0	Mucinous	MCN	MSKCC	MCN		Low
3	0	Mucinous	MCN	MSKCC	MCN		Low
3	0	Mucinous	MCN	MSKCC	MCN		Low
3	0	Mucinous	MCN	MSKCC	MCN		Mod
3	0	Mucinous	MCN	MSKCC	MCN		Low
3	0	Mucinous	MCN	MSKCC	MCN		Low
3	0	Mucinous	MCN	MSKCC	MCN		Low
3	1	Non-mucinous	SC	MSKCC	SC	macrocystic	
3	1	Non-mucinous	SC	MSKCC	SC	macrocystic	
3	1	Non-mucinous	SC	MSKCC	SC	macrocystic	
3	1	Non-mucinous	SC	MSKCC	SC	macrocystic	
3	0	Non-mucinous	SC	MSKCC	SC	microcystic	
3	0	Non-mucinous	SC	MSKCC	SC	macrocystic	
3	1	Non-mucinous	SC	MSKCC	SC	microcystic	
3	0	Non-mucinous	SC	MSKCC	SC	macrocystic	
3	0	Mucinous	PanIN I/II retention	MSKCC	Retention cyst	PanIN I/II present	Mod
3	1	Mucinous	IPMN	MSKCC	IPMN, main duct	intestinal	Low
3	1	Mucinous	IPMN	MSKCC	IPMN	intestinal	Mod
3	1	Mucinous	IPMN	MSKCC	IPMN, branch duct		Mod
3	0	Mucinous	IPMN	MSKCC	IPMN, branch duct	gastric type	Mod
3	1	Mucinous	IPMN	MSKCC	IPMN, branch duct		Low

3	1	Mucinous	IPMN	MSKCC	IPMN	mixed gastric/intestinal type	Mod
3	1	Mucinous	IPMN	MSKCC	IPMN		Mod
3	1	Mucinous	IPMN	MSKCC	IPMN, mixed main/branch duct	gastric type	Mod
3	1	Mucinous	IPMN	MSKCC	IPMN, main duct	Carcinoma; intestinal	High
3	1	Mucinous	IPMN	MSKCC	IPMN, main duct	Carcinoma; intestinal	High
3	1	Mucinous	IPMN	MSKCC	IPMN	gastric type	Mod
3	1	Mucinous	IPMN	MSKCC	IPMN	gastric type	Low
3	1	Mucinous	IPMN	MSKCC	IPMN, carcinoma (cis)	gastric type, carcinoma in-situ	High
3	1	Mucinous	IPMN	MSKCC	IPMN, carcinoma; main duct	carcinoma	High
3	0	Mucinous	IPMN	MSKCC	IPMN, branch duct	gastric type	Mod
3	1	Non-mucinous	SC	MSKCC	SC	macrocystic	
3	1	Non-mucinous	SC	MSKCC	SC	microcystic	
3	0	Non-mucinous	SC	MSKCC	SC	macrocystic	
