# Supplementary material for: A Gastric Glycoform of MUC5AC Is a Biomarker of Mucinous Cysts of the Pancreas
Source: PLoS One. 2016 Dec 19;11(12):e0167070. doi: 10.1371/journal.pone.0167070 (PMC5167232; doi:10.1371/journal.pone.0167070)
Supplement: S2 Table — (PDF) [file pone.0167070.s004.pdf]

S2 Table. Reagent Information.

Antibodies				
**Name**	**Target**	**Clone**	**Source**	**Catalog #**
Anti A4GNT	A4GNT	Polyclonal	Sigma-Aldrich	HPA008017
Anti aGlcNAc	alpha-linked GlcNAc	HIK1083	Cosmo Bio	KAN-02101-96-EX
Anti BGH	Blood Group H antigen	87-N	Abcam	ab24222
Anti Endorepellin	Endorepellin	Polyclonal	R&D Systems	AF2364
Anti MUC5AC	MUC5Ac	45M1	Thermo Scientific	MS-145-P1ABX
Anti MUC5AC	MUC5Ac	45M1	AbD Serotec	1695-0128
Anti MUC5AC (Ab2)	MUC5Ac	2-11M1	Thermo Scientific	MA1-35704

Purified Glycoproteins		
**Name**	**Source**	**Catalog #**
Laminin	Sigma-Aldrich	L-6274
Fibronectin	Sigma-Aldrich	F-0895
Transferrin	Fitzgerald Industries	30C-CP4060
Fetuin	Sigma-Aldrich	F2379
Carcinoembryonic Antigen (CEA)	Fitzgerald Industries	30-AC32

Enzymes			
**Name**	**Target**	**Source**	**Catalog #**
PNGase F	N-glycans	New England Biolabs	P0704S
α2-3,6,8 Neuraminidase	α2-3, 2-6, and 2-8 sialic acid	New England Biolabs	P0720S
β(1-3,4)-galactosidase (Bovine)	Terminal β1-3, β1-4 galactose	ProZyme	GKX-5013
α-N-Acetylgalactosaminidase	Terminal αGalNAc	New England Biolabs	P0734S
β-N-acetylhexosaminidase (Jack Bean)	Terminal GalNAc and βGlcNAc	ProZyme	GKX-5003

Lectins				
**Name**	**Abbreviation**	**Primary Specificity**	**Source**	**Catalog #**
Erythrina cristagalli lectin	ECL	Terminal Galβ	Vector Labs	BK-3000
Griffonia simplicifolia lectin II	GSL-II	Terminal α,β-GlcNAc	Vector Labs	BK-3000
Helix aspersa agglutinin	HAA	GlcNAcα, GalNAcα	Sigma-Aldrich	L8764
Ricinus communis agglutinin I	RCA-1	Terminal galactose	Vector Labs	BK-1000
Sclerotium rolfsii lectin (recombinant)	SRL	Terminal GlcNAcβ or GlcNAcα	Wako	199-17271
Wheat germ agglutinin	WGA	GlcNAc > sialic acid	Vector Labs	B-1025
